# A Rare Case of Central Odontogenic Fibroma of the Mandible

**DOI:** 10.7759/cureus.48772

**Published:** 2023-11-14

**Authors:** Nikil Jain, Abhishek Dutta, Nishant Kumar, Anusila Dalapati, Aishwarya Sinha, Sudhir M Ramesh

**Affiliations:** 1 Oral and Maxillofacial Surgery, Awadh Dental College and Hospital, Jamshedpur, IND

**Keywords:** mandible tumor, enucleation and curettage, diagnosis and treatment, odontogenic tumour, central odontogenic fibroma

## Abstract

The benign tumor central odontogenic fibroma (COF) accounts for less than 1% of all the existing odontogenic tumors. The mandibular or maxillary cortical plate is seen to show asymptotic diversification. It has been characterized as a benign jaw neoplasm. Radiographically, it primarily manifests as a multilocular radiolucency. Histologically, it comprises fibroblasts and mature collagen fibers. The popular choice for the management of COFs is enucleation, followed by the extraction of associated teeth. COFs have maintained a track record of showing rare chances of recurrence following surgery. COF was detected in a 38-year-old female who had edema in the lower right front tooth region. The lesion was surgically removed, and a histopathological examination was performed. Many case reports of COF have been stated in the literature. This indicates that cases of COF are not a rare appearance.

## Introduction

A central odontogenic fibroma (COF) is considered to be an uncommon development of mature odontogenic mesenchymal cells. It is so scarce that reliable distributions of location, gender, and age cannot be ascertained [1]. Those observed have happened in each jaw over a wide age range, with no sex preferences recognized [2]. It appears as a radiolucent lesion that shows well-defined margins, either unilocular or multilocular. Radiolucency appears to be associated with the crown of an unerupted tooth in young individuals due to its growth in areas of developing teeth. It generates a painless enlargement that may displace or resorb tooth roots [3,4].

The COF is thought to be the periodontal membrane's counterpart to the peripheral odontogenic fibroma [5]. Indeed, the core odontogenic fibroma may develop from partially generated somatic mesenchyme, which would normally become the periodontal membrane. This origination, however, would not be from the odontogenic apparatus in and of itself but rather from the somatic mesenchymal cells influenced by the odontogenic apparatus [2]. The radical theory is that it emerges from the genuine odontogenic mesenchymal cells of the dental papilla, like the odontogenic myxoma. However, the rate of maturity of the mesenchyme and its invasive capability are different from those of myxoma. COF also has a restricted growth capacity and, hence, shows low chances of recurrence [2]. Herein, we aim to present a case of COF with observed morphological and histopathological results that coincide with similar cases reported in the literature to date. The patient's consent was obtained for uploading the pictures and for publication.

## Case presentation

**Figure 1:**
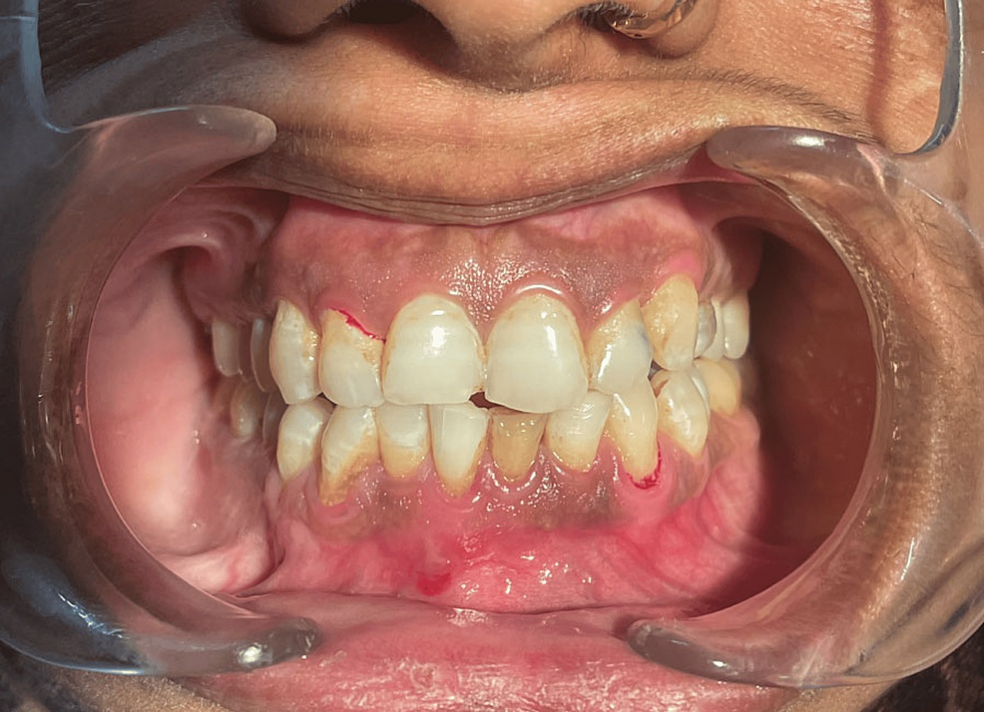
A 38-year-old female presented with painless enlargement related to the region of the lower front teeth

A 38-year-old female presented to the Department of Oral and Maxillofacial Surgery, Awadh Dental College and Hospital, Jamshedpur, India, with painless enlargement related to the region of lower front teeth (Figure 1). The patient first noticed this bulge about a year ago, which steadily grew in size. The patient was having pain while chewing and had no remarkable medical history.

Based on clinical, radiological, and histological results, COF was diagnosed, and the patient was counseled to undergo a long-term clinical investigation. After one year following surgery, there have been no signs of recurrence in the patient. The patient is still undergoing follow-up. An extraoral check confirmed the absence of edema. The temperature remained constant, with the color of the overlying skin unaltered. There were no palpable lymph nodes. The enlargement extended from the distal of the lower right canine to the mesial of the lower left first premolar on intraoral inspection. There was no indication of paresthesia. The lesion was solid and consistent. The lingual and buccal sulcus had been obliterated, and the overlying mucosa of that area was spotted with reddish and yellowish spots.

**Figure 2:**
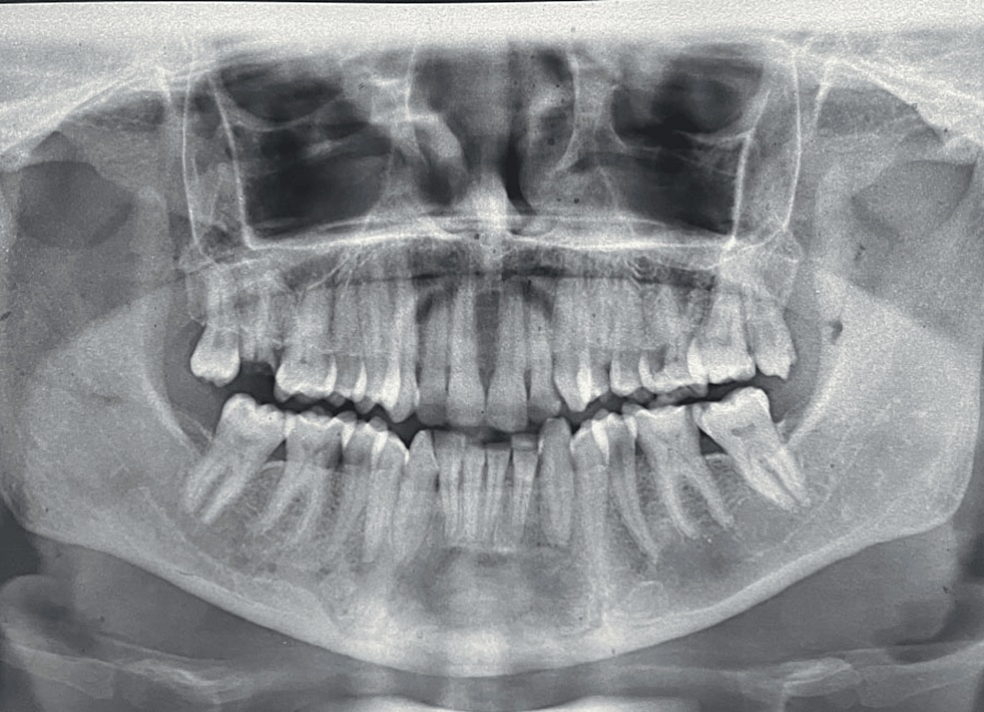
An OPG showing a massive multilocular radiolucent lesion of the anterior jaw

**Figure 3:**
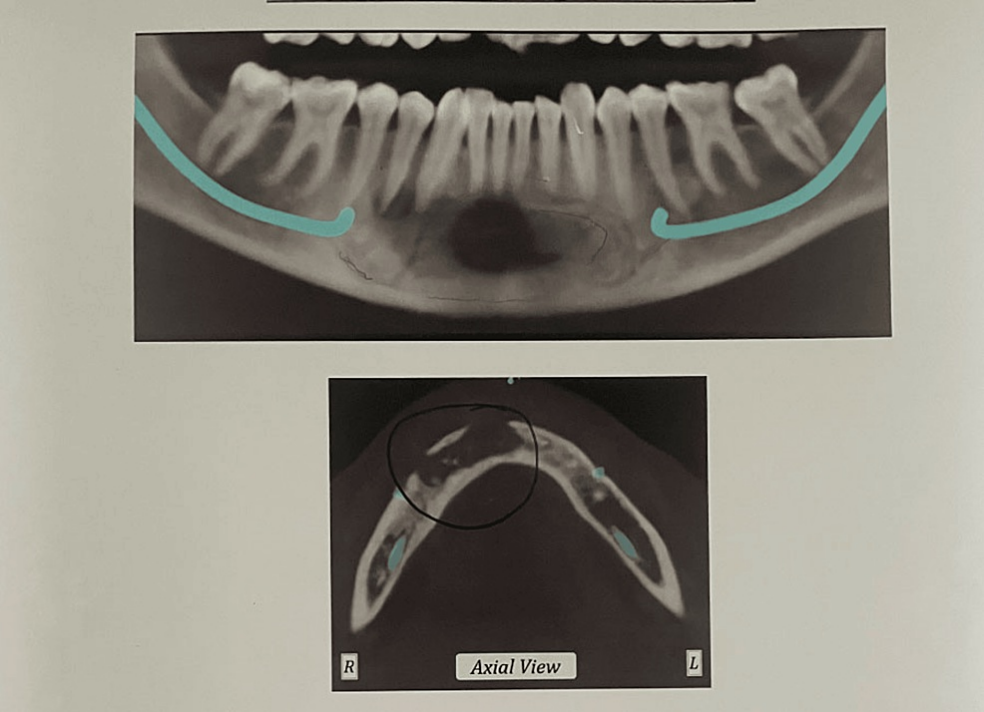
An OPG showing no sign of root resorption of the teeth associated with the radiolucent lesion

**Figure 4:**
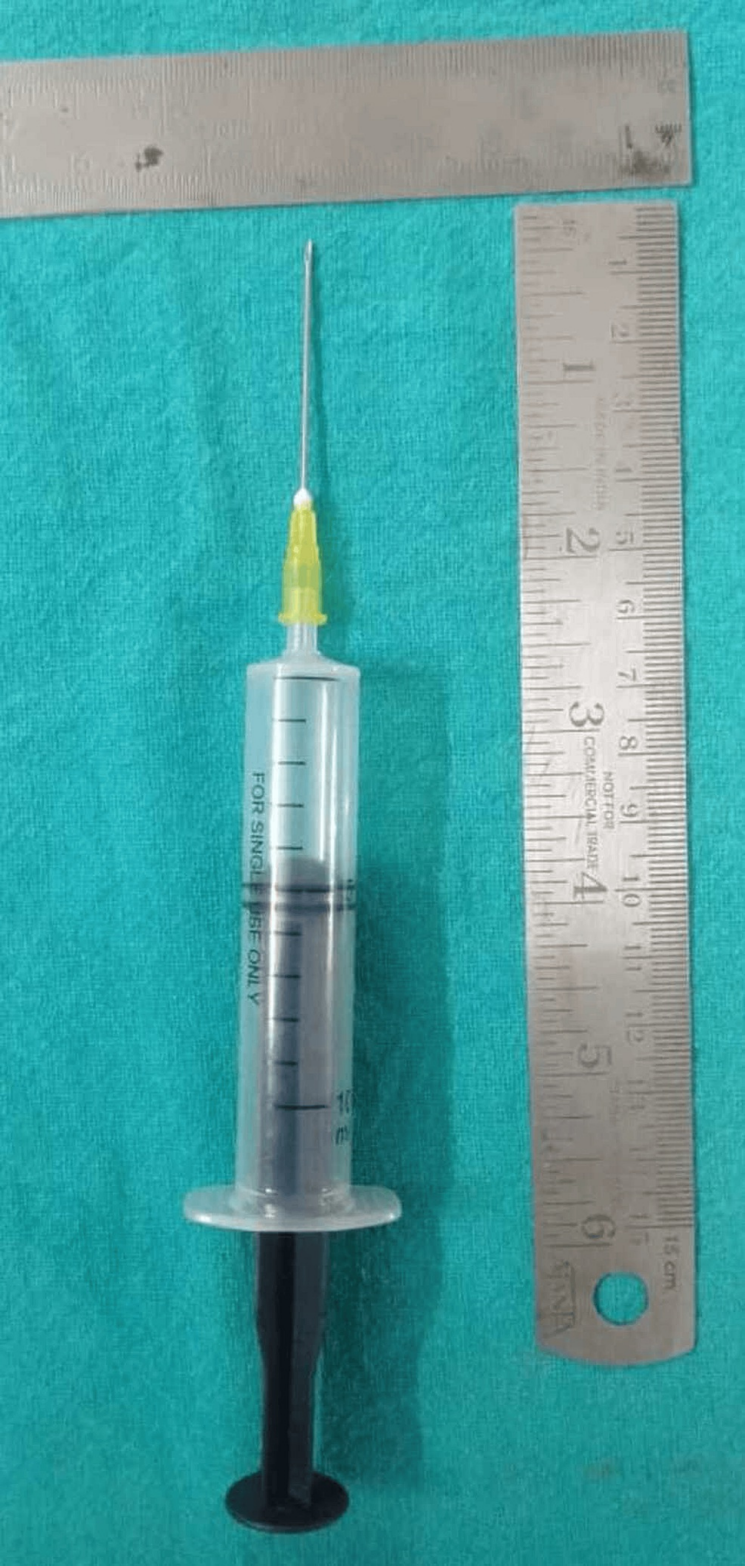
Needle aspiration was performed using a 16 gauge needle which yielded no results

A massive multilocular radiolucent lesion of the anterior jaw was shown by an orthopantomogram (OPG), extending from the distal of the lower canine in the right quadrant to the mesial surface of the lower left first premolar (Figure 2). There was no sign of root resorption (Figure 3). Needle aspiration was performed using a 16 gauge needle, which yielded no results (Figure 4). The differential determination of the lesion includes both the myxoma of odontogenic cells and ameloblastoma.

**Figure 5:**
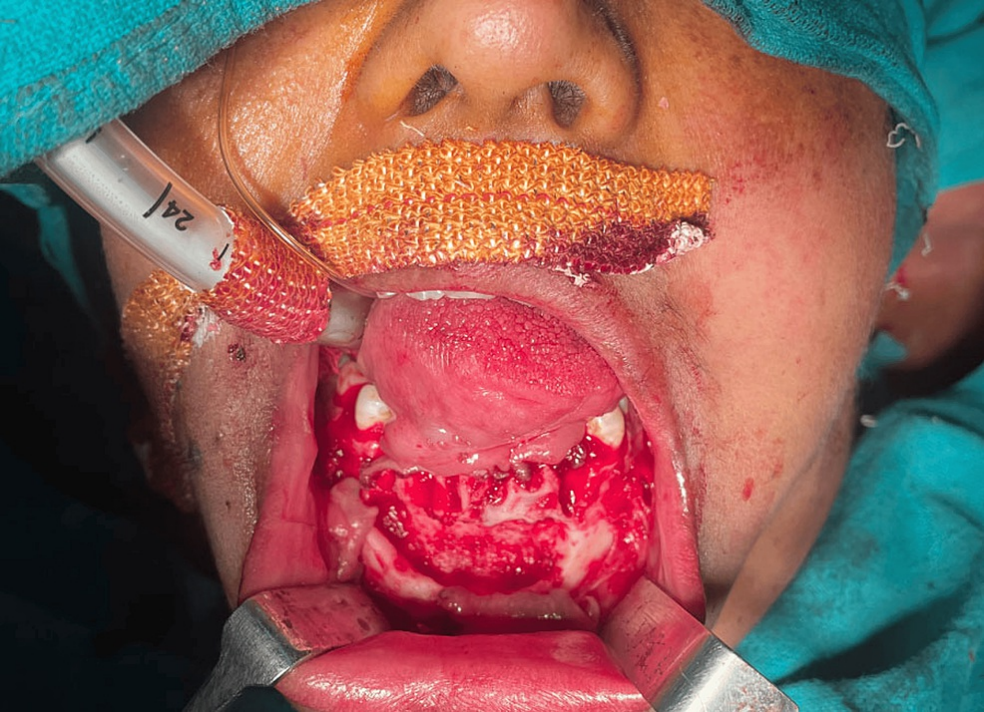
Under general anesthesia, the lesion was enucleated via the vestibular route

**Figure 6:**
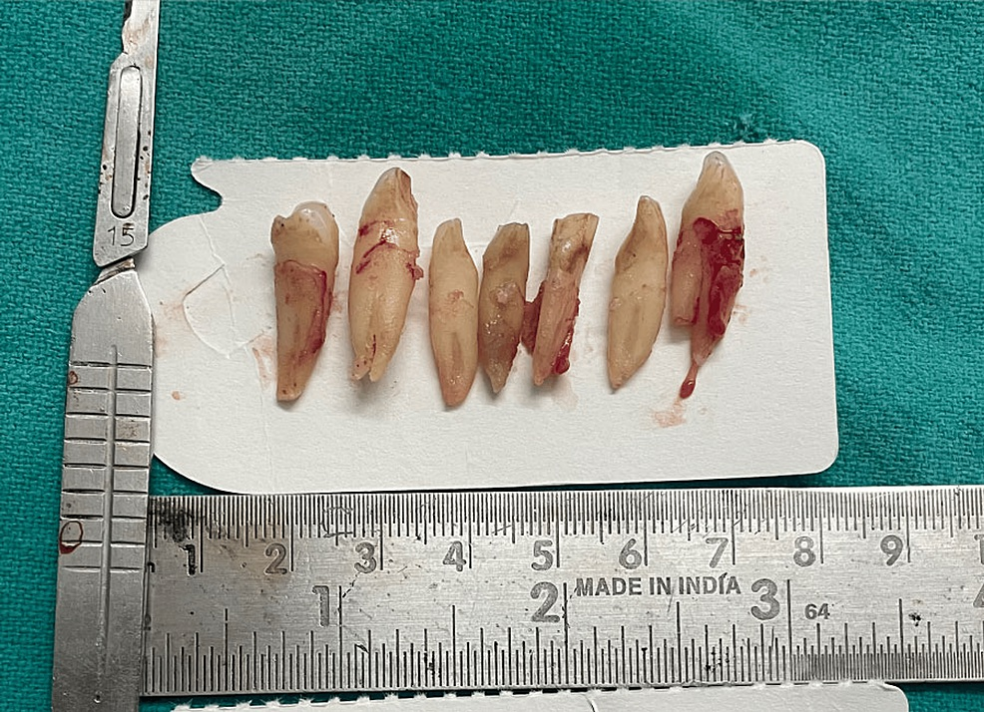
All of the lesion's embedded teeth were removed

Under general anesthesia, the lesion was enucleated via the vestibular route and sent for histological investigation (Figure 5). All of the lesion's embedded teeth were removed (Figure 6). We have planned for implants in the lower jaw once the healing is satisfactory.

**Figure 7:**
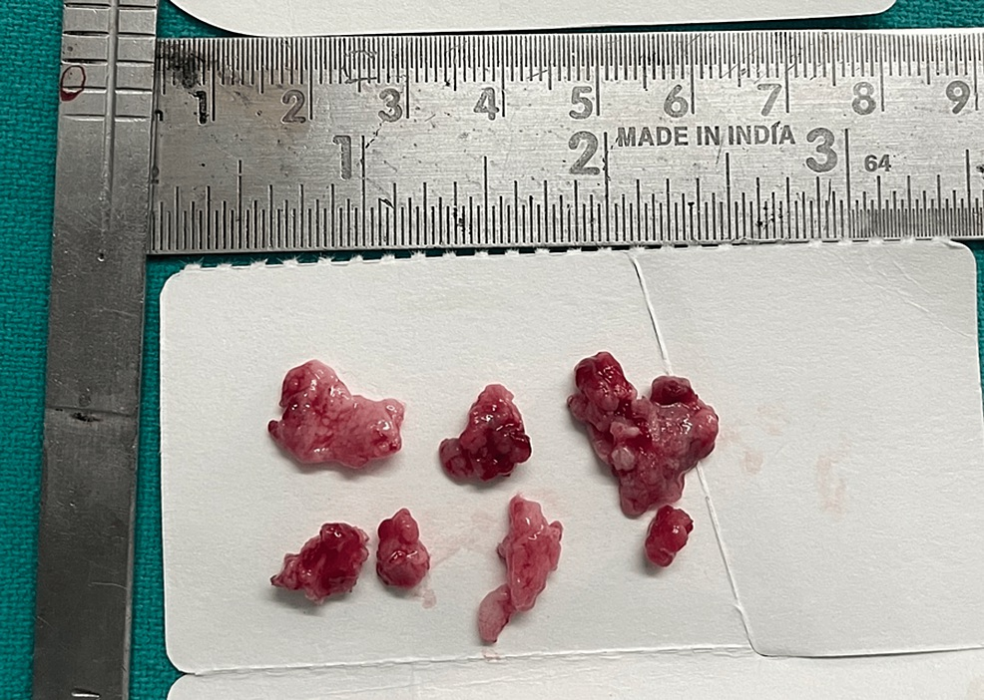
Resected lesion

**Figure 8:**
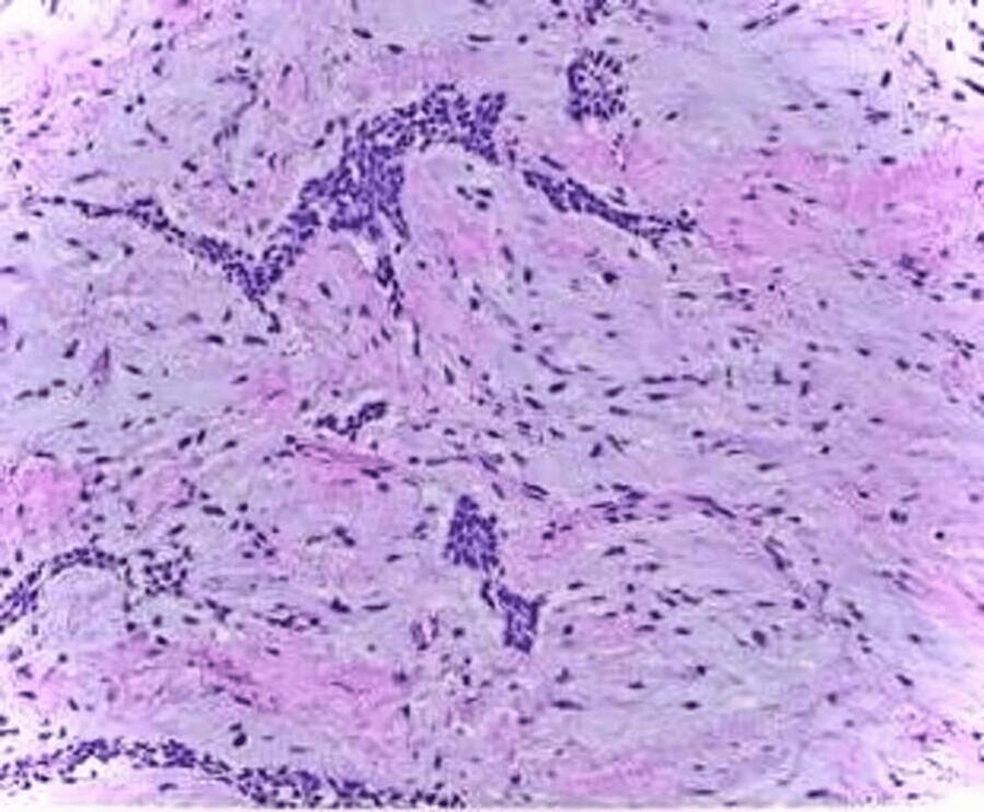
Histopathological picture of the specimen A lesional mass of cellular and fibrovascular connective tissue was discovered during histopathological analysis

The lesion can be seen in Figure 7. The tumor was 2 x 3 cm in size. A lesional mass of cellular and fibrovascular connective tissue was discovered during histopathological analysis. Numerous thin, long strands and nests of odontogenic rests were evident in the stroma. There was no evidence of bone growth or calcification. Inflammatory cell infiltrates were prominent in connective tissue with hemorrhagic regions (Figure 8).

## Discussion

COF has a slight female predilection as reported in our case and many other cases [6]. Generally, the cases occur in a mean age range of 34 years as seen in our case [6,7]. However, an unusual case of COF was seen in a 12-year-old boy who presented with asymptomatic gingival enlargement in the mandibular region [8]. Most of the cases are reported in the mandible [9,10] except for a few which may show up in the maxilla [11,12].

Wesley et al. [13] proposed the following criteria for diagnosing odontogenic fibroma in 1975. On clinical examination, the lesion is situated centrally in the jaws. It grows slowly and steadily resulting in painless cortical enlargement. Second, it can have a range of radiological characteristics, but the majority of cases (like odontogenic myxoma and ameloblastoma) are lesions that are multilocular and radiolucent in nature. It involves large parts of the jaws in the upcoming stages. In certain circumstances, the lesion is also associated with teeth that are misplaced and/or unerupted. Third, the predominant histologic feature is a tumor that is largely formed of mature collagen fibers with numerous fibroblasts that are intermingled. Small strands and/or nests of latent odontogenic epithelium are a variable characteristic. Fourth, the benign lesion responds very effectively toward surgical enucleation, with no propensity toward malignancy. By using these criteria, it was concluded that only seven true odontogenic fibromas and a single novel illustration were found in the mandible. This validates the tumor's rarity.

For most COFs, an incisional biopsy is recommended as the appearance indicates a more advanced disease [1]. Needle aspiration of the fluid in our study yielded no results. On the contrary, bloody and viscous tan material was obtained on a fine-needle-aspiration biopsy obtained in a case submitted by Santoro et al. [8]. After establishing the diagnosis, only a panoramic radiograph is sufficient for determining the treatment. Nah described a case of COF that appeared as a multilocular radiolucent lesion on a panoramic radiographic examination [14]. A literature review found 29 case reports of intra-osseous odontogenic fibroma, out of which 15 cases showed multilocular radiolucency while 12 cases showed unilocular radiolucency [15]. Our case showed a massive multilocular radiolucent lesion. On the contrary, the majority of COFs were found to be unilocular radiolucent lesions. However, they might also appear as multilocular lesions and might exhibit a mixed radiolucent-radiopaque appearance in rare cases [16]. The great variability in the radiologic appearance of the COFs means that it should be considered in the differential diagnosis of all radiolucencies found in the jaws.

Enucleation and curettage are used to treat COF [2]. They easily detach from the bony crypt and do not exhibit any bony invasion. The resulting bone space, which is mucosally closed, does not indicate a need for draining or packing [1]. In this scenario, the rate of recurrence is very rare. Dunlap and Barker reported two cases of maxillary odontogenic fibroma treated with curettage over a period of 9 and 10 years, respectively, with no recurrence [17]. Since a majority of the case reports studied to date showed no chance of recurrence [11,18], a similar treatment plan was followed in our case as well. As expected, no sign of recurrence was seen in our patient one year after the surgery.

However, certain cases of recurrence have been described. Jones et al. described a clinical scenario where the cancer returned 16 months following the surgical procedure [10]. Svirsky et al. have until now documented about 13% (in around 2 out of 15 patients) of the recurrence rate among the cases [18].

If a recurrence is noticed, Marx recommends reviewing both the initial pathology specimen and the biopsy material [1]. A fibromyxoma is an odontogenic myxoma with fibrous traits that was most likely misdiagnosed as a COF. It is possible that an ossifying fibroma with few bony cementum-like components was misdiagnosed as a COF with developed fibrous connective tissue and its own calcific deposits assumed to be dentin or cementum [19]. In 1983, Shafer et al. classified COF as an odontogenic fibroma, which is a unique neoplasm showing distinct clinical and histopathologic features, clearly distinguishing it from several other odontogenic tumors [2].

Gardner [8] sought clarification of previously known odontogenic fibroma lesions in 1980, categorizing them into three different categories (but presumably the lesions are related). First, the initial structure is a hyperplastic dental follicle. Second, the subsequent kind is a fibrous neoplasm with variable collagenous, fibrous connective tissue, and simple odontogenic epithelial nests. Third, a more sophisticated lesion is characterized by the cementum or dentine-like tissues showing dysplasia with variable degrees of WHO-type odontogenic epithelium. Gardner categorized the latter kind of tumor as odontogenic fibroma (WHO type) because it matches the calcifying odontogenic tumor (COT) identified by Pindborg in a WHO article published in 1971.

The calcifying odontogenic lesions differ from the non-calcifying odontogenic lesions in that this lesion has amyloid staining but is not similar to the odontogenic fibroma (WHO type). Over 80 people have described single instances or case series in English literature so far [11,16-21].

## Conclusions

Histopathological analysis (along with clinical and radiographic findings) is necessary for every specimen in order to reach a final diagnosis among suspected differential lesions. The great variability in the radiologic appearance of the COFs means that it should be considered in the differential diagnosis of all radiolucencies found in the jaws. Since COFs have shown very low recurrence rates, an incisional biopsy followed by enucleation and curettage can be considered the preferred choice of management. Many case reports of COF have been stated in the literature.
